# Toxicity and treatment discontinuation with adjuvant immune checkpoint blockade: a correspondence

**DOI:** 10.1097/JS9.0000000000003466

**Published:** 2025-09-09

**Authors:** Rui Zhong, Longjun Huang, Penglin Tang

**Affiliations:** a The First Clinical Medical College of Zhejiang Chinese Medical University, Hangzhou, China; b Zhejiang University of Technology, Hangzhou, China

*Dear Editor*,

I have carefully read the study by Zhao *et al*[1], published in the *International Journal of Surgery*. As a clinical oncologist with a research interest in immuno-oncology, I found this meta-analysis to be both timely and clinically relevant. The authors have undertaken a comprehensive evaluation of the safety profile of immune checkpoint inhibitors (ICIs) in the adjuvant setting, an area of growing importance as these agents are increasingly incorporated into standard treatment paradigms.

The strengths of this work are numerous. The inclusion of 38 randomized controlled trials with over 25 000 patients represents the largest and most up-to-date synthesis of evidence in this field. The dual approach of single-arm and pairwise meta-analyses allows for a nuanced understanding of both absolute incidence and relative risk increases associated with ICIs. The subgroup analyses by Immune Checkpoint Blockade（ICB） type, treatment strategy, tumor site, and trial design are particularly valuable, as they help clinicians contextualize the risks for specific patient populations. CTLA-4 blockade is associated with a significantly higher risk of treatment-related death, an important distinction that should guide clinical decision-making and trial design. Furthermore, the use of Grading of Recommendations Assessment, Development, and Evaluation (GRADE) methodology to assess the certainty of evidence adds rigor and transparency to the conclusions.

Despite the overall rigor of the study design, there are some limitations that warrant consideration in future studies. The reliance on trial data, while methodologically sound, may limit generalizability to real-world populations that include older patients, those with comorbidities, or those with prior autoimmune conditions[2]. Additionally, the relatively short follow-up in many adjuvant trials means that late-onset or chronic toxicities may be underreported[3]. Future updates of this meta-analysis could benefit from longer-term safety data as they become available.

Another point worth highlighting is the heterogeneity in reporting standards for immune-related adverse events (irAEs) across trials. While the authors made efforts to harmonize definitions, inconsistencies in grading and attribution remain a challenge in immuno-oncology meta-analyses. The application of a standardized reporting framework in future studies may enhance the comparability of safety data across studies.

Finally, although the meta-regression did not identify significant effects of age, sex, or ethnicity on toxicity risk, the available data may still be insufficient to detect subtler subgroup effects[4]. As adjuvant ICI therapy expands into broader populations, prospective studies with preplanned subgroup analyses will be essential.

In summary, Zhao *et al* have provided a robust and much-needed safety overview of adjuvant ICIs that will serve as a key reference for clinicians, researchers, and regulators. Their work underscores the need for careful patient selection, especially when considering CTLA-4-based regimens, and highlights the importance of multidisciplinary management for irAEs. I look forward to seeing future iterations of this analysis as more long-term data emerge.

## Data Availability

Not applicable.
